# Treatment of colitis by oral negatively charged nanostructured curcumin in rats

**DOI:** 10.1590/acb370602

**Published:** 2022-08-15

**Authors:** Lívia Medeiros Soares Celani, Eryvaldo Sócrates Tabosa Egito, Ítalo Medeiros Azevedo, Cláudia Nunes Oliveira, Douglas Dourado, Aldo Cunha Medeiros

**Affiliations:** 1 Fellow master degree. Universidade Federal do Rio Grande do Norte – Postgraduate Program in Health Sciences – Natal (RN), Brazil.; 2 PhD, full professor, chairman. Universidade Federal do Rio Grande do Norte – Laboratory of Dispersed Systems – Natal (RN), Brazil.; 3 PhD. Universidade Federal do Rio Grande do Norte – Health Sciences – Natal (RN), Brazil.; 4 PhD. Universidade Federal do Rio Grande do Norte – Pathology Department – Health Sciences – Natal (RN), Brazil.; 5 Fellow PhD degree. Universidade Federal do Rio Grande do Norte – Postgraduate Program in Health Sciences – Natal (RN), Brazil.; 6 PhD, full professor, chairman. Universidade Federal do Rio Grande do Norte – Nucleus of Experimental Surgery – Natal (RN), Brazil.

**Keywords:** Colitis, Therapeutics, Nanostructures, Curcumin, Mesalamine, Rats

## Abstract

**Purpose:**

To examine the effects of a negatively charged nanostructured curcumin microemulsion in experimental ulcerative colitis (UC) in rats.

**Methods:**

Four percent acetic acid was used to induce UC. The animals were treated for seven days and randomly assigned to four groups: normal control (NC), colitis/normal saline (COL/NS), colitis/curcumin (COL/CUR), and colitis/mesalazine (COL/MES). The nanostructured curcumin was formulated with a negative zeta potential (-16.70 ± 1.66 mV). Dosage of the pro-inflammatory cytokines tumor necrosis factor-α (TNF-α), interleukin 1-β (IL-1β), interleukin 6 (IL-6), and antioxidant enzymes (catalase, superoxide dismutase, and glutathione peroxidase), macro and microscopic evaluation of the colon tissue were analyzed.

**Results:**

The COL/CUR group had a higher level of antioxidant enzymes compared to the COL/MESgroup. The levels of TNF-α, IL-1β and IL-6 were significantly lower in the colonic tissue of the COL/CUR group rats, when compared to the COL/NS and COL/MES groups (p < 0.001). The presence of ulcers in the colonic mucosa in rats of the COL/NSgroup was significantly higher than in the COL/MES group (p < 0.001). In the NC and COL/CUR groups, there were no ulcers in the colonic mucosa.

**Conclusions:**

The nanostructured microemulsion of curcumin, used orally, positively influenced the results of the treatment of UC in rats. The data also suggests that nanostructured curcumin with negative zeta potential is a promising phytopharmaceutical oral delivery system for UC therapy. Further research needs to be done to better understand the mechanisms of the negatively charged nanostructured curcumin microemulsion in UC therapy.

## Introduction

Ulcerative colitis (UC) is a chronic disease, most common in people aged between 30 and 40 years old. It most commonly starts in the rectum, and over time it reaches other segments of the colon^1^. Owing to the chronic and intermittent characteristics of UC, treatment is long-lasting, using a combination or alternating medications. The objectives of therapy are to reduce symptoms and to induce cure, maintaining it as long as possible. The choice of therapy depends on the severity and complications^2^^,^ 3. In cases of mild to moderate UC, aminosalicylates (mesalazine or sulfasalazine) are the first line therapy^4^. When patients have moderate to severe UC, corticosteroids, immunomodulators, or biological therapies are usually required, alone or in combination, to avoid the risks^5^^-^ 8, all resulting in high annual treatment costs^9^. Immunomodulators include thiopurines, methotrexate and cyclosporine, while biologic ones include tumor necrosis factor-a (TNF-α) antagonists, integrin inhibitors and anti-interleukins.

Curcumin is a yellow powder extracted from the tuber of the *Curcuma longa*, originally from India and its geographic area. It has been commonly employed in Indian cuisine and traditional medicine of several countries^10^^,^ 11. Curcumin modulates multiple cells signaling pathways as a highly pleiotropic molecule. It is a polyphenol extensively studied for its effects as anti-inflammatory, antimicrobial, healing, hypoglycemic, antioxidant, neuroprotective, antineoplastic, etc. Several experimental studies and clinical trials have concluded that curcumin has great potential for the treatment of various diseases in humans^12^^-^ 14. Among the mechanisms of therapeutic action of curcumin, there are the suppression of activated B cell nuclear factor kappa-light-chain-enhancer and the suppression of TNF-α, interleukin 1-β (IL-1β) and interleukin 6 (IL-6)^15^. Due to these relevant activities, curcumin has potential for the treatment of UC^16^. Curcumin in clinical therapy is limited, because it is highly hydrophobic, has low intestinal absorption, limited biodistribution, rapid metabolism, rapid elimination, and presents low physicochemical stability^17^. Many curcumin formulations have been characterized and studied to improve these limitations, using temperature and polymeric micelles^17^^,^ 18.

For the formulation of curcumin used in this study, we opted for microemulsion, defined as an isotropic and thermodynamically stable system with nanoparticles ranging from approximately 10 to 50 nm^19^. Microemulsion systems (oil-water-oil) form spontaneously, without high-energy processes, and this is a difference when compared with other emulsified systems^20^. One of the key components in the preparation of microemulsions is the use of surfactants, responsible for the reduction of interfacial tension and spontaneous formation^21^. In the pharmaceutical field, its composition allows carrying different drugs with different polarities. Additionally, the nanometric particles size implies a large contact surface area, which allows for increased absorption and bioavailability^22^. Study by Zhao *et al*.^23^ concluded that proteins of the superficial cells of inflamed mucosa in mice colitis are positively charged. They treated mice with nanostructured nanozyme, negatively charged by oral administration, and preferentially it accumulated at inflamed mucosa.

Therefore, this study aimed to examine the effects of a negatively charged nanostructured curcumin microemulsion for the therapy of UC in rats.

## Methods

### Animals

Twenty-four male Wistar rats (*Rattus norvegicus*) weighing 275 ± 22 g were used. The study was performed at the Nucleus of Experimental Surgery, Universidade Federal do Rio Grande do Norte (UFRN), Natal, Rio Grande do Norte, Brazil, under control of room temperature (22°C), humidity (45-65%) and automatic light control. Rats were randomly allocated into four groups of six animals each. They were housed in rat cages with *ad libitum* access to food (Presence^®^) and water. Animal care was carried out in accordance with Brazilian Law no. 11,794/08, which deals with animal research. The research project was submitted to the institutional Ethics Committee on the Use of Animals and approved by the protocol no. 04/2020. All animals were acclimated for one week at the Nucleus of Experimental Surgery before the study started.

### Ulcerative colitis induction

UC was induced by colonic instillation of four percent acetic acid (AAC), purchased at Sigma-Aldrich (code A6283). The animals were sedated with an intraperitoneally (i.p.) injection of 7 mg/kg xylazine and 50 mg/kg ketamine. A liquid paraffin-lubricated polyethylene catheter with an internal diameter of 1 mm was introduced into the colonic lumen via the rectum, up to 8 cm proximal to the anus. Each rat was given 1 mL of 0.9 percent normal saline (NS) solution rectally, and abdominal palpation removed feces. After that, in the colitis groups, 1 mL of four percent AAC was slowly infused into the colon. To prevent the loss of the AAC solution, the rats were placed in the Trendelenburg position for 1 minute.

### Curcumin nanostructured microemulsion production

Sigma Chemical Co. provided the curcumin (C1386, lot MKCD2451, 75% purity) and Span^®^ 80 (St. Louis, MO, United States of America). Polyethylene glycol (PEG) 30 castor oil was provided as a gift from Oxiteno (Sao Paulo, SP, Brazil). Miglyol^®^ 812N (medium-chain triglycerides) was donated by IOI Oleochemical GmbH Pharma (Hamburg, Germany).

A nanostructured microemulsion loaded of curcumin was previously developed and characterized in the institutional Laboratory of Dispersed Systems (Department of Pharmacy, UFRN, Natal, RN, Brazil). Briefly, 15% of surfactant blend (PEG 30 Castor Oil/Span 80^®^; 8.8/1.2 w/w), 5% of Miglyol^®^ 812N, 80% of purified water and curcumin (20 mg/g oil) were weighed in a glass flask. These components were maintained for 10 minutes under high-pressure homogenization by the ultrasound probe Vibra-Cell 75041 (Farmingdale, NY, United States of America), 40% amplitude, 750 W, frequency 20kHz). At the end, a yellowish translucent final curcumin-nanostructurated microemulsion was achieved. The zeta potential and the size of the nanoparticles were determined. The formulation was then reproduced accordingly.

### Experimental design

Seventy-two h after the injection of four percent AAC, all rats were treated for seven days:

- The animals of the normal control group (NC) received 1 mL of 0.9% normal saline (NS), orally, by gavage;
- Colitis/control group (COL/NS) was treated with 1 mL of NS, by gavage;
- Colitis/curcumin group (COL/CUR) received 15 mg/kg of a single-daily dose of liquid curcumin nanostructured microemulsion (1 mL), orally, by gavage. This dosage was used by Sharma *et al*.^24^;
- Colitis/mesalazine group (COL/MES) received 100 mg/kg of mesalazine, orally, by gavage. Mesalazine was prepared with saline and administered once daily, 1 mL by gavage.

The animals were observed daily in polypropylene cages for seven days of treatment and weighed daily to assess weight variation.

### Macroscopic and microscopic examination of the colon

Twenty-four h after the last day of treatment, all animals were euthanized with an overdose of thiopental 100 mg/kg i.p. A median abdominal incision was performed, and all the colon (except the cecum) was resected, opened longitudinally, washed with saline and evaluated for the level of mucosal lesions.

### Macroscopy

Specimens were immediately examined. A surgical microscope (DF vasconcelos, São Paulo, Brazil, 10x magnification) was used. The damages were scored using a scale of 0–5 by a pathologist blinded to the groups. The score for macroscopic lesions of the colon was as follows:

- 0: no lesion;
- 1: mucosal hyperemia without ulcers;
- 2: linear ulcers without colonic stenosis;
- 3: linear ulcers plus single-area colon stenosis;
- 4: colon ulcers in multiple areas;
- 5: large ulcers in addition to necrosis and perforation.

### Microscopy

Rats’ colons were prepared for histopathological examination. The samples were fixed in 10% buffered formalin for 48 h and treated for 24 h in an automatic tissue processor. Histological sections were obtained at a thickness of 4µ, and stained with hematoxylin-eosin. The morphological analysis was performed under an optical microscope (Olympus Microscope CX41, Tokyo, Japan) by an experienced pathologist blinded to the groups. The following histopathological parameters and respective scores were analyzed:

- Acute inflammatory cells (AIC) – scores from 0 to 3;
- Chronic inflammatory cells (CIC) – scores from 0 to 3;
- Fibrin deposition (FD) – scores from 0 to 1;
- Submucosal edema (SE) – scores from 0 to 2;
- Epithelial cell necrosis (ECN) – scores from 0 to 3;
- Number of mucosal ulcers (MU): scores from 0 to 4.

Scanning of slides and production of photomicrographs were performed in the Aperio equipment (Leica, Germany).

### Examination of antioxidant enzyme parameters

Colon tissue samples (50 mg each) were homogenized in suitable buffers. We used the supernatant to measure the antioxidant enzymes superoxide dismutase (SOD), catalase (CAT), and glutathione peroxidase (GPx), using assay kits from ABCAM (Cambridge, MA, United States of America). Dosages were performed in duplicate following the manufacturer’s instructions.

### Cytokine assays by ELISA

To quantify cytokine expression in colonic tissue, 50 mg of colonic tissues from each rat were used. Extracts were obtained using guanidine-HCl and Tris-HCl (pH 8.0) with a protease inhibitor. The extracts were centrifuged to eliminate insoluble materials. The analyses were performed using enzyme-linked immunoassay (ELISA) kits (PeproTech, Ribeirao Preto, SP, Brazil), following the manufacturer’s instructions. TNF-α, IL-1β and IL-6 in colon tissue were measured at 450 nm in a microplate reader (Expert Plus, Nova Analitica, Sao Paulo, SP, Brazil), with all standards and dosages performed in duplicate.

### Statistics analysis

The Shapiro-Wilk and Kolmogorov-Smirnov tests evaluated the assumption of normality. The hypothesis of difference between groups was tested by using the analysis of variance (ANOVA), as well as the Kruskal-Wallis, Tukey’s and Dunnett’s tests. P < 0.05 indicated a statistically significant difference. All data were automatically analyzed by the statistical software Statistical Package for the Social Scienceds (SPSS)^®^ 20 (Chicago, IL, United States of America).

## Results

### Body weight

Weight gain was evidenced in only two groups (normal control rats treated with saline and COL/CUR when compared before (D0) and seven days after the interventions (D7). In the groups with colitis treated with normal saline (COL/NS) and with mesalazine (COL/MES), weight loss was observed (p = 0.001). Statistical significance was observed in all comparisons, as summarized in Table 1.

**Table 1:** Results of the body weight of the animals, comparing day zero (D0) and day 7 (D7) of the experiments#.

Time course	Group				p-value*
	Normal control	COL/NS	COL/+CUR	COL/MES	
D0 (g)	246.5 ± 5.96§	256.8 ± 10.67w	192.7 ± 6.5§w	230.8 ± 5.98§w	< 0.001
D7 (g)	252.7 ± 9.46§	237.7 ± 4.23w	202.5 ± 16.18§w	221.5 ± 7.89§w	< 0.001
Gain/loss (%)	2.5% ± 1.8%§	-7.4% ± 2.3%§w	5.2% ± 1.6%w^Y^	-4.0% ± 2.2%^Y^	0.001

# Mean ± standard deviation; COL: colitis; NS: normal saline; CUR: curcumin; MES: mesalazine;

* analysis of variance and Tukey’s test;

**,§,w,Y data on the same line followed by identical signs means a significant difference between groups (p < 0.001).

### Histopathological examination

Table 2 indicates that the infiltration of acute inflammatory cells was significantly higher in the COL/NS and COL/MES group rats compared to the COL/CUR group rats (p < 0.001).

The level of mucosal ulcers (MU) in the group treated exclusively with acetic acid (COL/NS) was significantly higher (p = 0.001) compared to the mesalazine group (COL+MES). In the normal control and COL/CUR group rats, colonic ulcers were not found. These data are summarized in Table 2.

**Table 2:** Descriptive and inferential statistics of the histopathological examination.

Variable	Group				p-value*
	NC	COL/NS	COL/CUR	COL/MES	
AIC	0.00 (0.0-0.25)	3.00 (2.0-3.0)	1.50 (0-2.0)	3.00 (2.75-3.0)	0.007
CIC	0.50 (0-1.0)	2.00 (1.75-2.0)	0.00 (0.0-0.0)	2.00 (2.0-3.0)	< 0.001
SE	0.00 (0.0-0.0)	1.00 (1.0-1.25)	1.00 (0.75-1.0)	1.00 (0.75-1.0)	0.002
ECN	0.00 (0.0-0.0)	0.00 (0.0-2.25)	0.00 (0.0-0.0)	0.00 (0.0-1.5)	0.207
MU	0.00 (0.0-0.0)	3.00 (1.75-4.0)	0.00 (0.0-0.0)	2.50 (0.75-4.0)	0.001

AIC: acute inflammatory cells; CIC: chronic inflammatory cells; SE: submucosal edema; ECN: epithelial cell necrosis; MU: mucosal ulcers; NC: normal control; COL: colitis; NS: normal saline; CUR: curcumin; MES: mesalazine; median (interquartile deviation);

* Kruskal-Wallis p-value.

**Figure 1:**
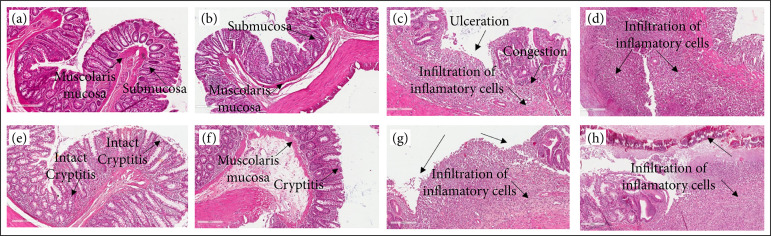
Histopathology of the colon, two images per group. Lesions are indicated by arrows. (**a** and **b**) Normal mucosa with regular structure from the normal control group rats. (**c** and **d**) There are ulceronecrotic inflammation and abscesses from the colitis/normal saline group rats, in both images. (**e** and **f**) These images show that the colitis microscopic status in colitis/curcumin group was significantly recovered. Criptitis and submucous edema are observed. (**g** and **h**) Two representative images from the colitis/mesalazine group rats show mucosal ulcers, areas of necrosis, vascular congestion, andinflammatory cells. Hematoxylin-eosin staining, ×200 magnification. Photomicrographs obtained using the Aperio, Leica, Germany. Scale bar 200 µm.

Figure 1 characterizes the histopathological features of colonic mucosa of rats subjected to acetic acid enema and respective treatments.

**Figure 2:**
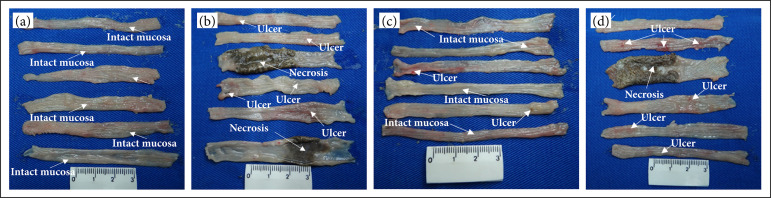
Representative macroscopic images of six fresh colon 8-cm samples for every group after treatments. Lesions are indicated by arrows. A surgical microscope (DF vasconcelos, São Paulo, Brazil ×10) was used. **(a)** Normal control group. The mucosa is completely normal in all samples. **(b)** Rats with colitis and treated with normal saline. Multiple ulcerations, edema, and areas of necrosis are seen. **(c)** The images of colitis rats treated with curcumin are similar to the normal control rats. **(d)** Colitis rats treated with mesalazine. Multiple ulcerations of different sizes, edema, necrosis, and dilation of the colonic wall.

Figure 2 shows macroscopic images of the colonic mucosa of six rats from each group. In the NC group (Fig. 2a), the mucosa is completely normal in all colons. In those rats of COL/NS group, there are areas of edema and mucosal ulcers of different sizes in all specimens (Fig. 2b). In two of them, well-defined dark areas of necrosis and dilation of the colonic wall are observed. In the COL/CUR group rats (Fig. 2c), the colonic mucosa was similar to the mucosa of the normal control group rats, but sparse areas of edema and inflammatory reaction are shown. In colonic specimens from animals treated with acetic acid and mesalazine (COL/MES group), the mucosa exhibits multiple ulcerations of different sizes and edema. In one of the colons, there is an area of intense inflammation and necrosis of the mucosa, with dilation of the colonic wall (Fig. 2d).

### Antioxidant enzymes

The catalase, SOD and GSH-px enzymes were detected in the colonic tissue at higher levels in the normal control group rats, in comparison to the groups of animals with colitis, submitted to the respective treatments (p < 0.001). When curcumin microemulsion was added to the treatment of the colitis animals (COL/CUR group), tissue levels of SOD, catalase, and GSH-px were higher than in the COL/NS group (p < 0.001). The rats from group COL/MES had higher levels of antioxidant enzymes in comparison to the COL/NS group rats. However, the levels were lower than in the COL/CUR rats (p < 0.001) (Table 3).

**Table 3:** Descriptive results from the analysis of the dosage of antioxidant enzymes and respective inferential statistical tests#.

Variables	Groups				
	NC	COL/NS	COL/CUR	COL/MES	p-value$
Catalase (μmol/mg)	98.7 ± 9.2*	36 ± 5.6*	69 ± 6.0*	45 ± 5.2*	< 0.001
SOD (μmol/mg)	67.3 ± 7.0**	12 ± 3.2**	38 ± 4.1**	24 ± 3.7**	< 0.001
GSH-px (μmol/mg)	89.8 ± 8.5§	25 ± 5.1§	57 ± 6.3§	36 ± 4.4§	< 0.001

# Mean ± standard deviation; SOD: superoxide dismutase; GSH-px: glutathione peroxidase; NC: normal control; COL: colitis; NS: normal saline-0.9%; CUR: curcumin; MES: mesalazine;

$ p-value from analysis of variance and Tukey’s test;

*,**,§ values on the same line followed by the same sign mean significant differences between groups (p<0.001).

### Cytokine dosage

The levels of TNF-α, IL-1β and IL-6 in the colonic tissue of COL/CUR group rats were significantly lower than in the COL/NS and COL/MES groups (p < 0.001). Tissue cytokine levels in the COL/MES rats were significantly lower than in the COL/NS group rats (p < 0.001) (Table 4).

**Table 4:** Effect of curcumin and mesalazine on TNF-α, IL-1β and IL-6 levels in colon tissue#.

Cytokines	Group				p-value*
	NC	COL/NS	COL/CUR	COL/MES	
TNF-α (pg/mL)	40.3 ± 2.42§	436.2 ± 6.11§	170.5 ± 5.01§	185.7 ± 8.96§	< 0.001
IL-1β (pg/mL)	21.0 ± 1.90*	117.7 ± 3.20*	28.7 ± 1.51*	40.3 ± 2.80*	< 0.001
IL-6 (pg/mL)	20.3 ± 1.86§	89.8 ± 4.26§	55.5 ± 2.59§	67.0 ± 2.19§	< 0.001

# Mean ± standard deviation; NC: normal control; COL: colitis; NS: normal saline-0.9%; CUR: curcumin; MES: mesalazine;

* p-value of analysis of variance and Tukey’s test.

§,* p < 0.01, COL/CUR *vs*. NC, COL/NS, COL/MES groups.

Values on the same line with similar symbols mean significant differences between groups (p < 0.001); TNF-α tumor necrosis factor; IL: interleukin.

## Discussion

Although several drugs and combinations of them are being used to control UC, there is currently no permanent curative treatment for this disease. As for oral UC therapy, one of the strategies is the use of drug-loaded nanoparticles that can be transported through the gastrointestinal tract and released into the colonic lumen^25^. When drug treatment fails, in about 35% of patients, surgical treatment will be indicated. Considering a difficult-to-control disease, a search for new treatment options is needed^26^.

Curcumin has rapid metabolism, chemical instability, high hydrophobicity, and low intestinal absorption, resulting in poor effects in the treatment of UC^27^^,^ 28. Nanostructured systems have been used to carry a series of drugs with high potential for therapeutic applications. They can solubilize molecules with different properties, resulting in a safe biocompatible matrix^29^. The formulation used in this study was a yellow, translucent, stable, nanostructured curcumin microemulsion. The cells easily internalize nanostructured particles with biocompatible characteristics^29^.

It has been demonstrated that inflamed mucosa also has a high level of positively charged proteins. So, if the treatment is performed with a negatively charged nanostructured curcumin system, it should preferentially be attracted to the inflamed colonic mucosa^30^. In inflamed tissues, there are positively charged phosphoethanolamine and aminoarabinose, that are essential for the defense mechanisms^30^. Among the proteins that most contribute to the positive charge of inflamed cells, the histones are positively charged nuclear proteins. Their interaction with cell membranes is heavily dependent on positive charge^31^^,^ 32.

Zeta potential (ZP) is the electrostatic parameter in the electrical double layer that surrounds nanoparticles in solution. This is the most commonly used technique to measure the surface charge of nanoparticles^33^. In this study, we used a nanostructured curcumin microemulsion formulated and characterized in one of our laboratories (Laboratory of Disperse Systems) with ZP -16.70 ± 1.66 mV. We believe that the association of positively charged proteins in the inflamed cells of the colonic mucosa with the use of a nanostructured microemulsion of curcumin with negative ZP may have influenced the favorable outcome of the treatment on our UC model. By the way, Sharma *et al*.^24^ used an animal colitis model and quantitatively evaluated the absorption of curcumin nanoparticles in the digestive tract organs after oral administration. They demonstrated a high concentration of curcumin nanoparticles in the inflamed colon tissues, confirming great penetration and retention.

A possible etiology of UC is oxidative stress, under the effect of reactive oxygen species, such as hydrogen peroxide, hydroxyl radical, and superoxide anion. Oxidative stress induced by reactive oxygen stress exacerbates the inflammatory reaction producing positive feedback, inducing tissue damage^34^. Curcumin, a polyphenol from the extract of *C. longa*, has many anti-inflammatory actions^35^. CAT, GPx, and SOD are vital antioxidant enzymes, which are important biomarkers for oxidative stress, present in UC^36^^,^ 37. In this investigation, we observed that the treatment with nanostructured curcumin contributed to the elevation of the anti-inflammatory enzymes CAT, GPx, and SOD on the colonic tissues. Therefore, we can infer that the oral use of nanostructured curcumin, resulting in higher levels of the antioxidant enzymes, when compared to groups of rats treated with NS and MES, had a protective effect against colonic tissue inflammation.

In the pathogenesis of inflammatory bowel diseases, including UC, various immune cells are actively involved. Curcumin affects the function, differentiation, and maturation of dendritic cells, promoting the induction of intestinal T cells with a hyporesponsive phenotype, and the inhibition to present antigens^38^. Curcumin treatment reduced the expression of TNF-α, IL-1β and IL-6 in the colonic tissues, corroborating the findings of Burge *et al*.^37^. The administration of acetic acid to untreated animals significantly increased the level of TNF-α (436.2 ± 6.11 pg/mL), producing a pro-inflammatory cytokine storm. On the other hand, the animal group treated with curcumin nanostructured microemulsion had a significant reduction in the tissue levels of TNF-α (170.5 ± 5.01 pg/mL), as well as IL-1β and IL-6, compared to the colitis treated with NS, and to the COL/MES group rats. In addition, the weight gain of rats treated with curcumin showed positive results. Some other experimental studies have been published using plant extracts and drugs for the treatment of UC, emphasizing other mechanisms as inhibiting the secretion of IL-13, increased immunoexpression of NF-kB in rectum, etc.^38^^,^ 39. The histopathologic and macroscopic validations of the scores used in the present study were based on previous methods^40^.

The characterization of nanostructured systems, including curcumin microemulsion, has been studied at the Laboratory of Dispersed Systems, UFRN, Natal, RN, Brazil, and at other centers, supporting the discussion of its use in translational and clinical research. Characterization data have included stability, solubility, spectral analysis, conductivity, nanoparticle size, pH, biocompatibility, cytotoxicity, and ZP^41^^,^ 42.

Some reports support the viability of the acetic acid in the colitis model in rats, suggesting that this model can be successfully used to examine experimental UC^43^^,^ 44, as it was observed in this study. An attempt was made to use curcumin in its free form in an additional group. However, the high hydrophobicity of curcumin did not allow it. This fact could be interpreted as a limitation of this study.

## Conclusions

These findings suggest that nanostructured microemulsion formulation of curcumin, orally used, positively influenced the therapy of UC in rats. The results also suggest that this negatively charged nanostructured curcumin carrier may be considered as a promising phytopharmaceutical delivery system for UC therapy. Further research is needed to better understand the mechanisms of the effects of nanostructured curcumin microemulsion in the treatment of UC.
